# The Enigma of Big Tau Exon 4a: Genomic Architecture, Biophysical Identity, and Unique Evolutionary Mechanisms

**DOI:** 10.3390/biom16081215

**Published:** 2026-08-20

**Authors:** Itzhak Fischer

**Affiliations:** Department of Neurobiology and Anatomy, Drexel University College of Medicine, 2900 Queen Lane, Philadelphia, PA 19129, USA; if24@drexel.edu

**Keywords:** MAPT, tau isoforms, alternative splicing, tau aggregation, exonization, intrinsically disordered region, vertebrate evolution, axonal cytoskeleton

## Abstract

The microtubule-associated protein tau, encoded by the MAPT gene, serves as a major component of the neuronal cytoskeleton, facilitating the assembly, stabilization, and spatial organization of microtubules. Much of the work on tau has focused on the low-molecular-weight (LMW) isoforms abundantly expressed in the central nervous system (CNS) and their pathological aggregation in tauopathies. However, a different variant known as “Big tau”, present in the peripheral nervous system (PNS) and selective CNS regions has distinct structural and functional properties and offers a unique perspective on protein evolution. Big tau is characterized by the inclusion of a large, alternatively spliced insert termed exon 4a, which expands the protein’s projection domain by approximately 250 amino acids and increases the molecular weight to 90–110 kDa. The evolutionary trajectory of exon 4a presents a fascinating enigma that challenges conventional models of protein conservation. Across the vertebrate phylogeny, spanning from fishes, amphibians and birds to mammals, the primary amino acid sequence of exon 4a exhibits extreme divergence, often reaching background levels of identity when comparing distant classes. In contrast, the physical length of this domain remains remarkably stable, hovering around the 250-amino acid mark regardless of the species. This pattern suggests that the selective pressure acting on Big tau is not directed toward specific sequence motifs or functional domains, but rather toward the biophysical properties and physical dimensions of the domain. Here, we posit that exon 4a evolved as an essential molecular spacer optimized for the structural demands of long-projection neurons and high-caliber axons as well as a protective structure for the pathologic aggregation of tau. The paper examines the genomic architecture and biophysical identity underlying the stable-size and low sequence identity of exon 4a, presenting two evolutionary mechanisms as working hypotheses: a Prototype Model of neutral drift of an ancient insert, and an Independent Exonization of convergent recruitment of non-coding DNA by transposable elements or intron retention. Finally, we emphasize the need for additional experimental work in vitro and in vivo to resolve unanswered questions about the structure of the 4a exon, the physiological role Big tau and its potential insight into tauopathies therapeutics.

## 1. Introduction

**Figure 1:**
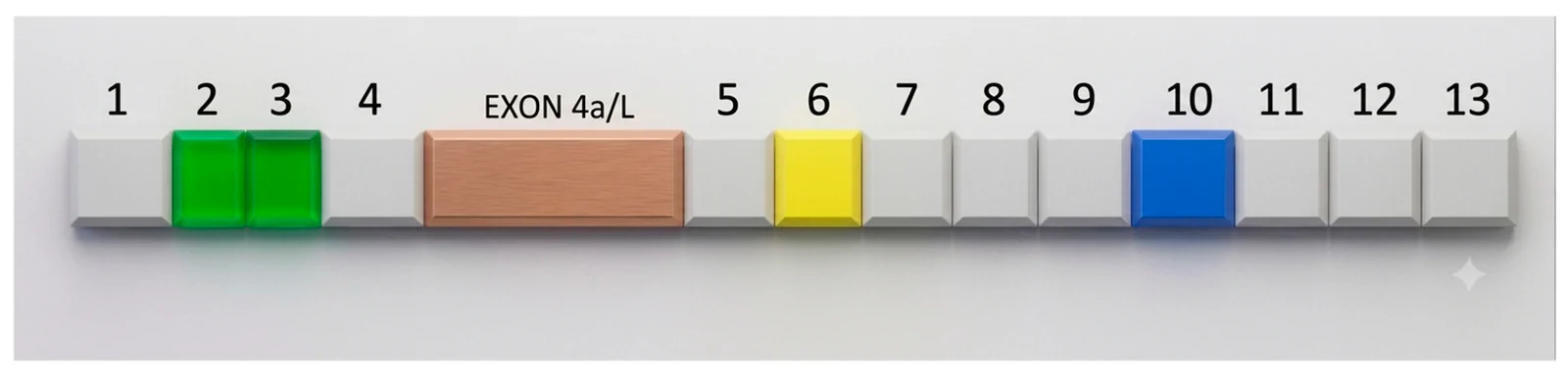
Exon structure of MAPT. The figure shows a schematic representation of MAPT exon structure (not to scale) where exons 2/3 (green) and exon 10 (blue) are alternatively spliced to form the 6 known isoforms of tau. Exon 4a and 4a-L (brown) together with exon 6 (yellow) are alternatively spliced to form Big tau. All together, these exons form the N-terminal domain and the 4 microtubule binding domain (exons 9–12) as described in details [3].

The human MAPT gene is located on chromosome 17q21.31 and is characterized by a complex structure comprising up to 16 exons [1,2]. The complexity of tau protein function is achieved through alternative splicing of several key exons, primarily exons 2 and 3 at the N-terminal and exon 10 in the C-terminal as part of the microtubule-binding domain (MTBD) [3]. In the human CNS, these events generate six canonical isoforms [4,5]. However, the inclusion of exon 4a introduces an entirely different scale of structural expansion (Figure 1). Exon 4a is the largest exon within the MAPT locus, spanning over 1 kilobase of genomic DNA [1]. Its insertion between exons 4 and 5 (hence 4a) results in a dramatic extension of the N-terminal projection domain, the portion of the protein that projects away from the microtubule surface into the surrounding cytoplasm [6]. What is remarkable about the 4a exon is that along the vertebrate phylogeny the primary amino acid sequence of exon 4a exhibits extreme divergence, while the length of this domain remains stable at around the 250-amino acid as shown in Table 1. Specifically, while the 4a sequence remains conserved among primates, it shows about 50% identity among other mammals and almost background levels at non-mammals [7].

While LMW tau is ubiquitous in the brain [8], Big tau is the primary isoform in the peripheral nervous system (PNS), cranial motor nuclei, and specific CNS populations that project to the periphery, such as spinal motor neurons as well as the cerebellum [9]. This spatial compartmentalization is accompanied by tight developmental control; neurons initially express LMW tau during early maturation and neurite outgrowth, switching to Big tau only as axons reach their targets and transition to a stabilized, mature state [10,11,12].

The structure of exon 4a is defined by its classification as an intrinsically disordered region (IDR) unlike structured domains that fold into defined three-dimensional shapes, exon 4a remains highly flexible, with a computational disorder prediction of approximately 96% [13]. This inherent lack of structure allows the domain to adopt different conformations, behaving like a random coil that occupies a large hydrodynamic volume allowing them to be involved in a multitude of processes [14,15,16]. A defining biophysical feature of exon 4a is its high acidity (negative charges) and hydrophilicity. The domain is enriched with acidic amino acids (glutamate and aspartate), resulting in a pronounced net negative charge at physiological pH. In humans, the ratio of negative to positive residues in the 4a sequence is 1.30, significantly higher than the 1.11 ratio found in the remainder of the LMW tau protein [13]. This charge profile appears to be one of the main features conserved across the vertebrate phylogeny, with acidic-to-basic ratios ranging from 1.3 in humans to as high as 2.3 in amphibians. The Kyte–Doolittle hydropathy scores for exon 4a remain consistently negative across all vertebrate species, indicating a robust hydrophilic nature that favors solvent exposure (Table 2). This high solubility and charge density are consistent with the domain’s physiological role; they create a strong electrostatic repulsion between adjacent tau molecules and microtubules, effectively transforming the protein into a so called “polyelectrolyte brush” or “entropic bristle”.

Exon 4a-L represents an extended variant of the standard exon 4a that defines the Big tau isoform. While canonical exon 4a encodes approximately 250 amino acids, the 4a-L variant encodes an elongated segment of about 355 amino acids [7]. Structurally, it maintains overall hydrophilic and polar properties, but its extended sequence introduces minor hydrophobic patches and a less uniform charge distribution, which may slightly alter its spacing efficiency and self-association tendencies compared to the shorter 4a isoform [13]. The 4a-L exon was originally identified in dysregulated contexts such as cancer and tumorigenic cells [17] but also described in transcriptomic and proteomic observations in several mammalian species including humans [7,18].

In this review we propose that the principal evolutionary function of Big tau is the generation of a long-range entropic and electrostatic exclusion zone that physically shields axonal microtubules and suppresses pathological tau-tau interactions.

## 2. Functional Hypotheses

The structural features of the 4a exon suggest two functional hypotheses; the Spacer Hypothesis related to properties of peripheral axons and the Protective Hypothesis related to shielding tau from aggregation.

### 2.1. The Spacer Hypothesis: Structure of the Axonal Cytoskeleton

**Figure 2:**
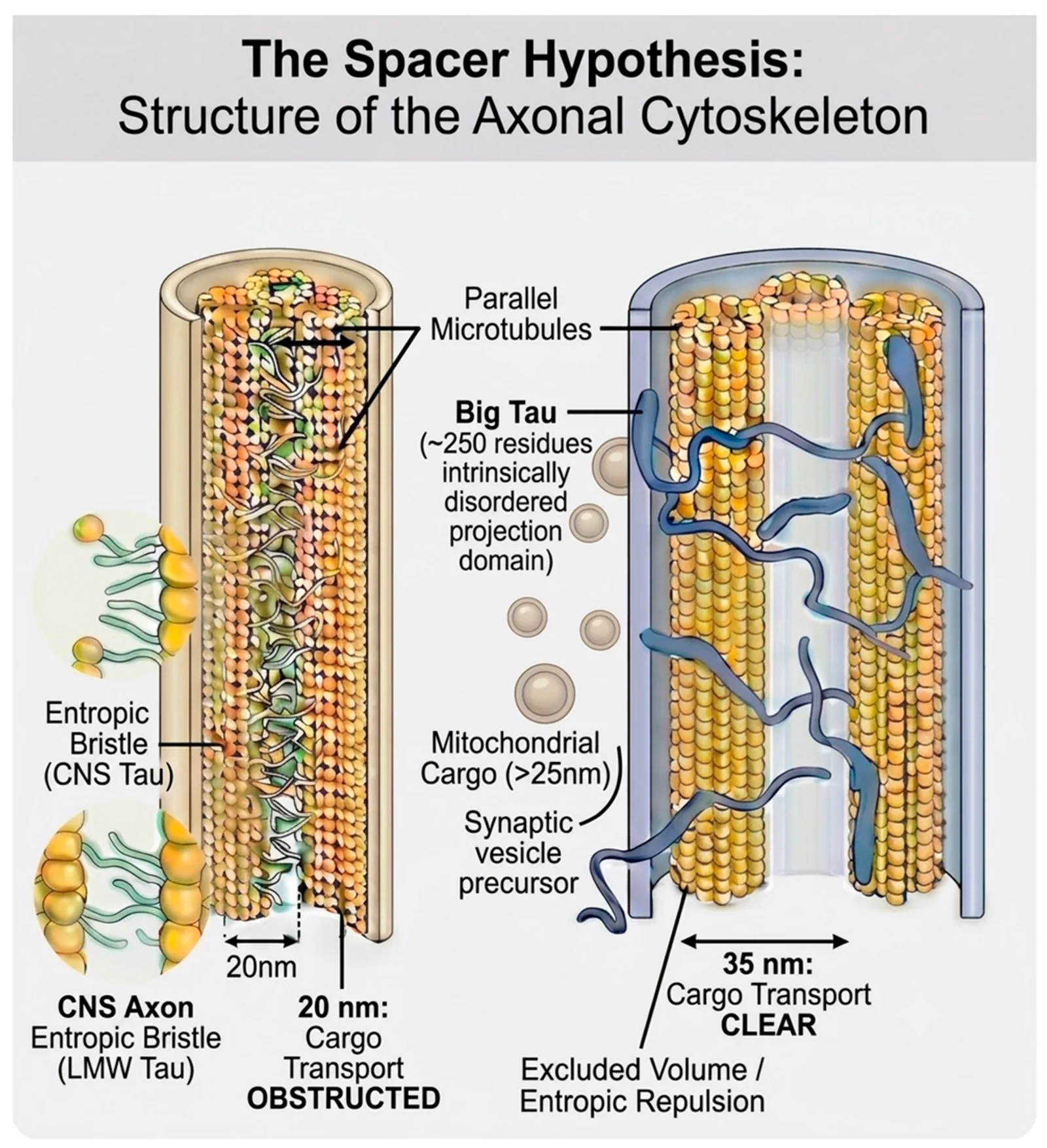
The Spacer Hypothesis. This figure illustrates the functional consequences of distinct tau isoforms on axonal architecture and transport. Left panel shows a CNS axon containing LMW tau. The smaller projection domain of LMW tau provides an approximately 20 nm inter-microtubule spacer, which may impede larger mitochondrial cargo. It highlights the “entropic bristle” nature of the LMW tau projection domain and its specific spatial effect. Right panel illustrates a PNS axon where the inclusion of the Big tau-specific exon 4a significantly extends the projection domain to ~250 residues to 35 nm inter-microtubule spacer. It shows that this increased reach (with a radius of gyration, R_G_ of 6 nm) effectively acts as an excluded volume or entropic repulsion, doubling the inter-microtubule space to 35 nm. This wider spacing can facilitate bidirectional long distant transport of peripheral neurons.

The functional explanation for the evolution of Big tau lies in the physical organization of the axonal structure. In the complex environment of a peripheral high-caliber axon, microtubules must be bundled into parallel arrays with precise inter-microtubule spacing to facilitate the bidirectional transport of organelles and vesicles (Figure 2). Tau functions as an entropic bristle, where the thermal fluctuations of its unstructured projection domain create a repulsive force that keeps adjacent microtubules separated. The length of the projection domain is the primary determinant of this spacing [19,20]. Experimental data from Sf9 cells overexpressing various tau isoforms demonstrate that LMW tau induces an inter-microtubule spacing of approximately 20 nm. In contrast, Big tau, with its expanded exon 4a insert, increases this spacing to approximately 35 nm [21]. This 15 nm increase is functionally significant in the context of axonal transport. High-caliber peripheral axons are engaged in long distance transport, including large mitochondrial networks and extensive synaptic vesicle precursors, which need an efficient mechanism to sustain this activity. By maintaining a length of ~250 amino acids, Big tau provides increased spacing that can facilitate the transport along microtubules preventing these cargoes from colliding with or being blocked by the dense microtubule network. The conservation of size across vertebrates reflects a physical requirement for a 35 nm gap in the most demanding neuronal transport environments.

The relationship between the length of exon 4a of about 250 residues and its physical reach is governed by the scaling laws of polymer physics. For a disordered polypeptide in the aqueous cytosol, the radius of gyration (R_G_) scales as R_G_ α N^0.6^ [22]. For a chain of 250 amino acids, the theoretical R_G_ is approximately 5.5 to 6.7 nm as a minimum, which does not account for the electrostatic stretching that can changes the scaling closer to 1.0. Because Big tau projects from the microtubule surface, this radius defines the “excluded volume”-a region where other proteins or organelles are sterically hindered from entering. The selection for a stable length of ~250 amino acids is essentially selection for a consistent volume of entropic repulsion, ensuring that the axonal “highway” remains clear of debris and roadblocks [23]. Altogether Big tau can also contribute to the mechanical resilience of axons with respect to axonal stretch tolerance and mechanical compliance and may serve as “molecular shock absorber” for large-caliber or mechanically stressed axons. Another element of the unique biophysical properties of Big tau is the slower diffusion compared to LMW tau calculated by the size differences to be 20–30% slower. It may be an adaptation to projecting neurons because it keeps Big tau distributed in a more locally persistent way along long axons rather than allowing rapid redistribution or ectopic accumulation.

An important parallel to the proposed spacer function of Big tau is found in the biology of neurofilament (NF) side arms which are critical determinants of axonal caliber and conduction velocity [24]. The C-terminal projection domains of the NF-H and NF-M subunits extend outward from the filament core as long, intrinsically disordered side arms that generate steric and electrostatic repulsive forces between adjacent neurofilaments [25]. These side arms behave as polyelectrolyte brushes, creating intermolecular spacing that helps maintain axoplasmic organization and mechanical stability. The physical principles governing NF side-arm function closely resemble the proposed entropic bristle model for Big tau exon 4a. Both systems rely on elongated intrinsically disordered regions enriched in charged residues, both generate long-range excluded-volume interactions, and both regulate cytoskeletal spacing within large-caliber axons.

### 2.2. The Protective Hypothesis: Exon 4a as a Shield Against Aggregation

Beyond its mechanical role as a spacer, Big tau exhibits a remarkable resistance to the pathogenic processes that lead to tauopathies [13]. In Alzheimer’s disease and related disorders, LMW tau dissociates from microtubules, misfolds, and aggregates into toxic neurofibrillary tangles which accumulates in the brain [26,27]. LMW tau which is an intrinsically disordered protein without a defined 3D structure can fluctuate over a wide range of conformations which are susceptible to aggregation into amyloid fibrils as their hydrophobic stretches are not buried inside a protein core [16]. Crucially, the peripheral nerves and brain regions where Big tau is the dominant isoform are highly resistant to these pathologies [9].

The aggregation of tau is nucleated by two hexapeptide motifs located within the second and third microtubule-binding repeats: PHF6 and PHF6* [26,28,29]. These motifs have a high propensity to form β-sheet structures, which are the building blocks of amyloid fibrils. Molecular modeling and in vitro assays suggest that the massive, acidic projection domain provided by exon 4a sterically and electrostatically “shields” these motifs [13].

**Figure 3:**
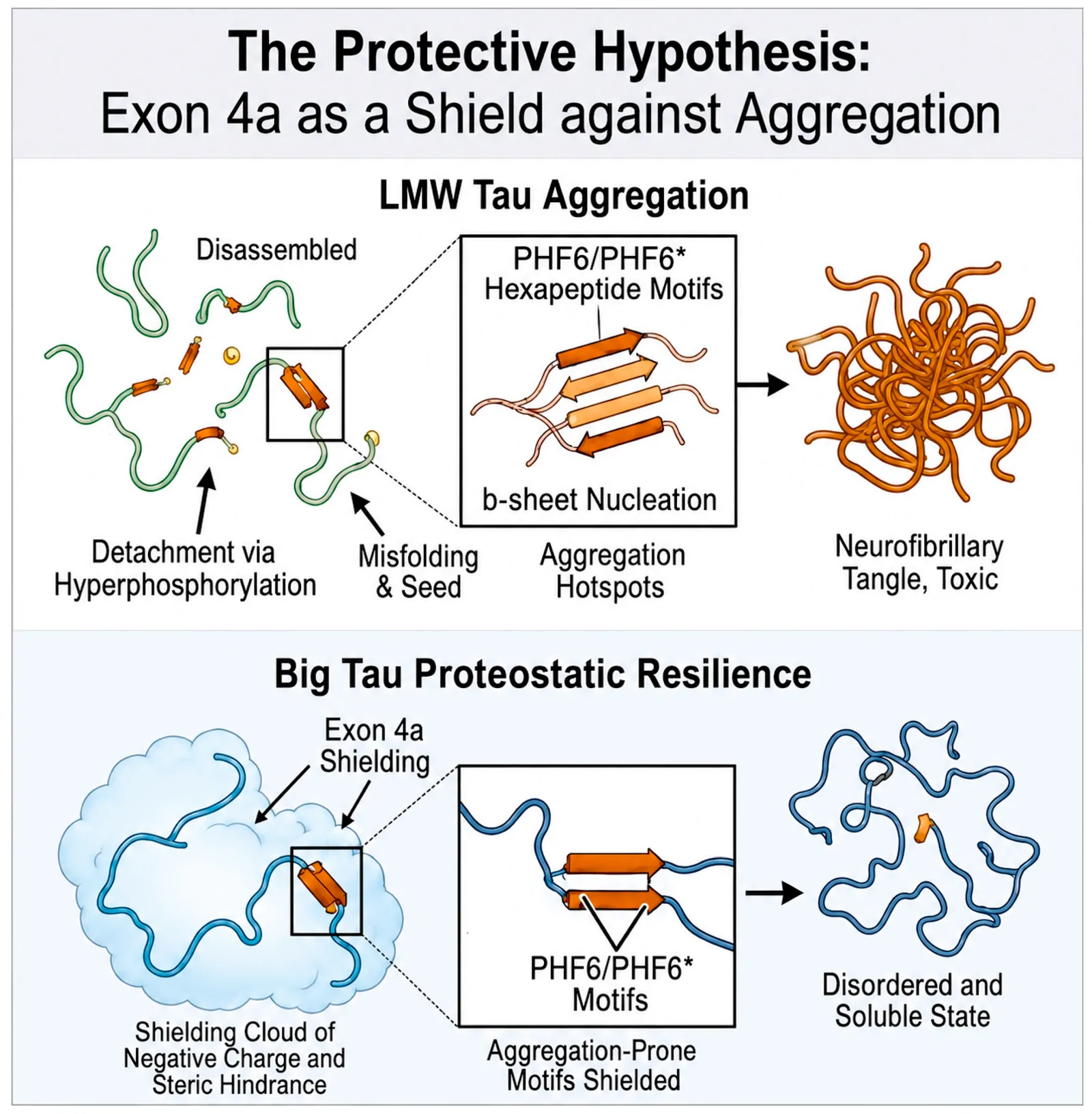
The Protective Hypothesis. The figure shows the differential aggregation propensities of LMW and Big tau. The top panel describes the classic pathological pathway: (1) LMW tau is hyperphosphorylated, causing detachment from microtubules; (2) the disassembled protein misfolds, exposing hexapeptide motifs (PHF6/PHF6*); (3) these “aggregation hotspots” nucleate cross-β sheet structures; (4) resulting in the formation of toxic amyloid fibrils and neurofibrillary tangles. The bottom panel, “Big Tau Proteostatic Resilience,” shows how the large, acidic, hydrophilic, and disordered exon 4a domain acts as a dynamic “proteostatic shield”. By utilizing entropic forces and dynamic negative charge, exon 4a sterically and electrostatically obscures the PHF6/PHF6* hotspots, preventing seed formation and amyloid nucleation. This unique biophysical shielding effect maintains Big tau in a soluble, disordered state, explaining the remarkable resilience of Big-tau-expressing peripheral neurons.

The volume occupied by the 250-amino acid disordered insert creates a dynamic “cloud” of negative charge and steric hindrance around the MTBD. This shielding limits the exposure of the aggregation-prone “hotspots”, effectively preventing the inter-molecular interactions necessary for the protein to seed and elongate into fibrils. Thus, this protective mechanism is largely independent of the specific amino acid sequence of exon 4a, as long as the domain maintains its size and negative charge density (Figure 3). Consequently, Big tau has a significantly lower propensity to form β-sheets (13.14%) compared to LMW tau (17.33%), contributing to the proteostatic resilience of Big-tau-expressing neurons. Big tau may also suppress pathological phase transitions by maintaining tau molecules below the critical concentration needed for fibril nucleation. Specifically, Liquid–liquid phase separation (LLPS) is an intermediate step that can lower the barrier to pathological aggregation by creating a concentrated, multivalent environment [30,31]. Such a model can help explain how soluble tau can transition into the insoluble neurofibrillary tangles seen in Alzheimer’s disease and other tauopathies [32]. Big tau may modulate tau LLPS by reducing the concentration of phase-separation-competent tau available for condensate formation, which could in turn diminish the propensity for downstream aggregation.

LMW tau contains over 80 potential phosphorylation sites, and hyperphosphorylation at these residues is the primary trigger for its detachment from microtubules and subsequent aggregation [2,33,34]. In contrast, the human exon 4a insert, despite its large size, is predicted to contain fewer phosphorylation sites—only two in most bioinformatics models [35]. This relative “phosphorylation immunity” ensures that the expanded projection domain remains in a highly disordered, soluble state even under conditions of cellular stress that would normally cause LMW tau to collapse and misfold.

## 3. The Evolutionary Enigma: Contemplating on the Origins of Size Conservation

**Figure 4:**
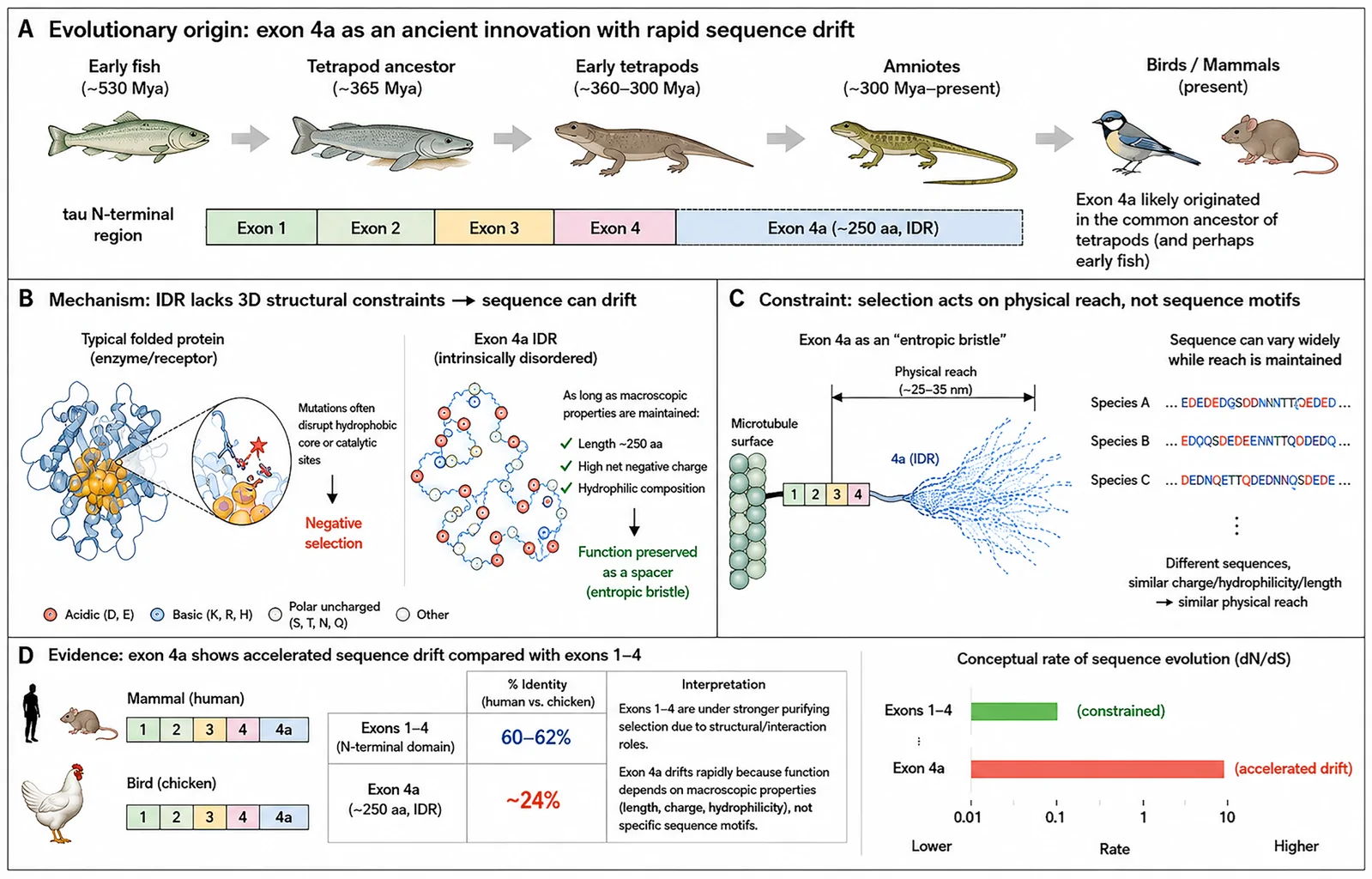
Prototype model. The figure illustrates the hypothesis that exon 4a originated early in vertebrate evolution and subsequently underwent rapid sequence divergence because it functions as an intrinsically disordered region (IDR) lacking strict three-dimensional structural constraints. Exon 4a is proposed to be conserved primarily through macroscopic biophysical properties, including length (~250 amino acids), high negative charge, and hydrophilic composition, which maintain its role as an “entropic bristle” spacer extending from the microtubule surface. Comparative analysis demonstrates accelerated sequence drift in exon 4a relative to the more conserved N-terminal tau exons (1–4), supporting the idea that selective pressure acts on physical reach and spacing rather than precise amino acid sequence motifs.

One of the main mysteries of Big tau is the discrepancy between its extreme sequence drift and its stable physical dimensions. While the MTBD and N-terminal functional domains of tau show upwards of 90% sequence identity between mammals and birds [36,37], the identity of exon 4a drops to background levels (15–24%) across the same evolutionary distance. Nevertheless, the exon’s length consistently remains near 250 amino acids (Figure 4). This suggests that evolution has optimized the reach of the domain, the physical distance it can project into the cytoplasm rather than its specific biochemical interactions. Thus, the evolutionarily conserved biophysical solution is implemented through sequence-divergent intrinsically disordered domains.

### 3.1. The Prototype Model: Neutral Drift of an Ancient Insert

The “prototype model” suggests that exon 4a is an ancient vertebrate innovation that originated in the common ancestor of all tetrapods (and perhaps early fish) [7,38]. In this model shown in Figure 4, the absence of a defined three-dimensional fold allowed the sequence to mutate rapidly, as there was no requirement to maintain precise inter-residue contacts for stability. Selection acted only on the macroscopic properties: a length of about 250 residues, a high negative charge and hydrophilic composition of amino acids [13].

**The mechanism:** Because the projecting domain of the 4a domain is an intrinsically disordered region (IDR), it lacks the defined 3D structural constraints present, for example, in enzymes or receptors [14]. In a typical folded protein, mutations often disrupt the hydrophobic core or catalytic sites, leading to negative selection [39]. In exon 4a, however, as long as the critical biophysical properties such as the net negative charge, hydrophilic structure and length are maintained, the primary amino acid sequence can drift almost randomly without losing its function as a spacer. Similar evolutionary behavior has been described for other IDRs such as the C-terminal domain of p53 and low-complexity regions in RNA-binding proteins, where sequence divergence is high but functional properties like disorder and charge distribution are conserved [15,39].

**The Constraint:** Selection acts on the physical reach rather than specific sequence motifs [22]. Evolution appears to tolerate substantial sequence variation, provided the total count of amino acids and the ratio of acidic-to-basic residues remain stable to preserve the “entropic bristle” function.

For example, the N-terminal domain (exons 1–4) of tau shows 60–62% identity between mammals and birds, while exon 4a in those same species drops to ~24%. This suggests exon 4a has an “accelerated drift” rate because it lacks the sequence-specific binding roles found in the earlier exons [13].

### 3.2. Independent Exonization: Convergent Recruitment of Non-Coding DNA

**Figure 5:**
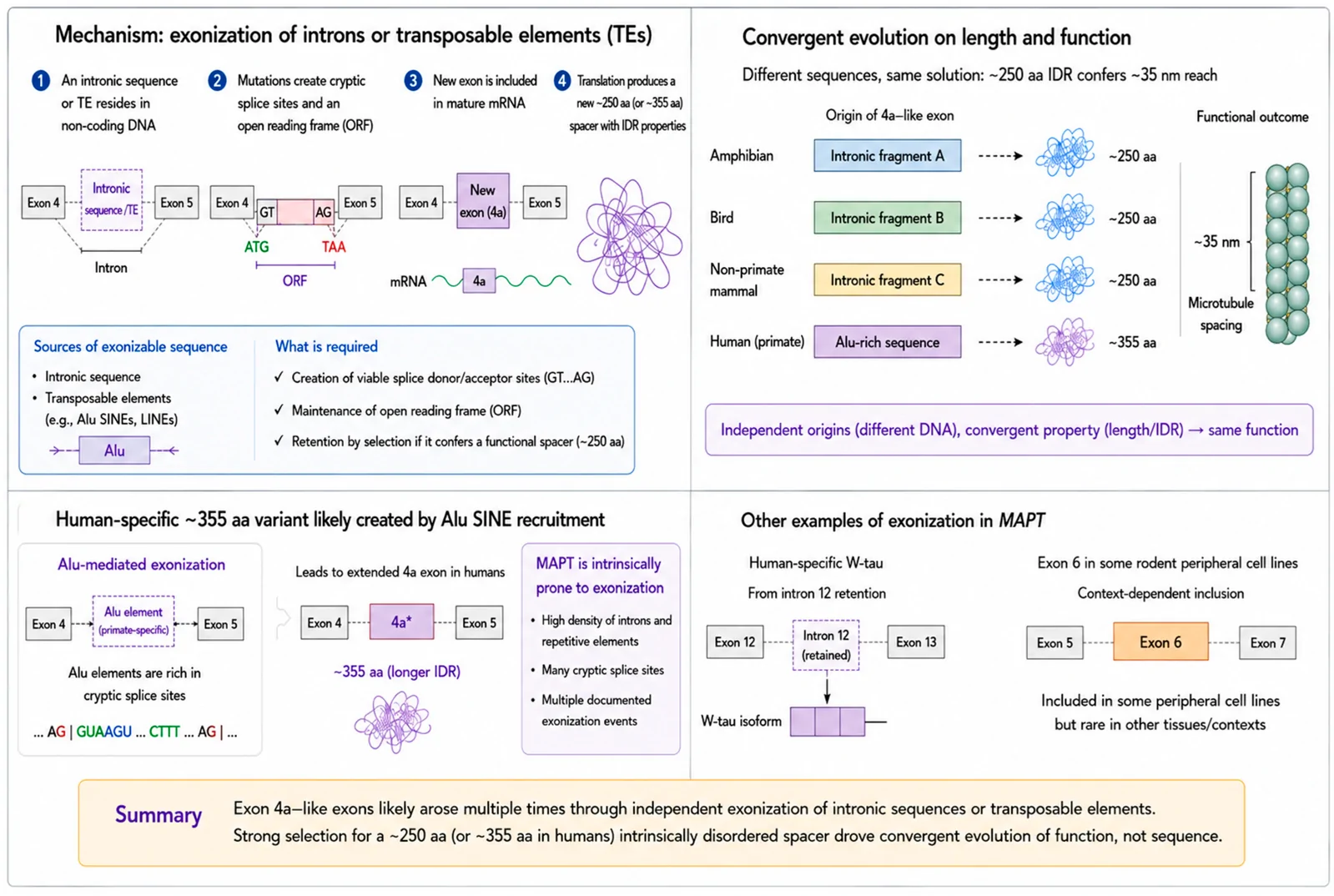
Independent exonization. Independent exonization model. The figure presents an alternative model in which exon 4a regions evolved independently in multiple vertebrate lineages through exonization of intronic or transposable element-derived sequences. Mutations generating cryptic splice sites and open reading frames allowed previously non-coding DNA to become incorporated into mature MAPT transcripts. Different lineages have recruited distinct genomic sequences that converged on a similar intrinsically disordered spacer length (~250 amino acids) capable of producing the microtubule spacing required for mature peripheral axons. The figure also highlights the human-specific ~355 amino acid variant, potentially generated through recruitment of primate-specific Alu SINE elements, and summarizes additional exonization events within the MAPT locus, including W-tau and exon 6 variants.

An alternative, and more complex model involves independent exonization (Figure 5). This model proposes that exon 4a-like variants evolved multiple times in different lineages through the recruitment of non-coding intronic sequences or transposable elements (TEs) [7,13]. Exonization occurs when intronic sequences acquire mutations that create viable splice sites and a continuous open reading frame (ORF), allowing them to be integrated into mature mRNA transcripts [40,41,42]. This model suggests that the selective pressure for a 250-amino acid spacer was so great to retain the function of Big tau that different vertebrate lineages independently acquired non-coding sequences (introns) or transposable elements (TEs) to serve this role.

**Mechanism:** Exonization occurs when an intronic sequence or a retrotransposon (like an Alu element) acquires a mutation that creates a viable cryptic splice site and a continuous open reading frame (ORF). This allows previously noncoding DNA to be integrated into the mature mRNA transcript as a new coding exon.

**Convergent Evolution:** The genomic material from which exon 4a emerged may differ across lineages. Under this model, a bird might have exonized one intronic sequence, while an amphibian exonized a completely different one. Both converged on the 250-residue length because it provided the specific 35 nm microtubule spacing required for their mature peripheral nerves. Humans can also express a unique 355-amino acid variant which was found so far only in prostate cancer cells lines [17]. This extension likely resulted from the recruitment of primate-specific Alu SINE elements, which are rich in cryptic splice sites. The MAPT gene appears inherently prone to these events. Other examples include the human-specific W-tau resulting from intron 12 retention [3,43,44], exon 3 retention and splicing in of exon 6, which is included in some rodent peripheral cell lines but rare in other contexts. This is evidence of evolutionary and regulatory plasticity of MAPT, consistent with exonization and with a sequence context that tolerates major alternative splicing changes. It suggests that the surrounding region of 4a exon was permissive enough for exonization to emerge more than once during evolution. A summary and comparison of the 2 mechanistic models that are consistent with low sequence identity and relatively stable size is shown in Table 3.

## 4. Developmental Switches and Tissue-Specific Splicing

The expression of Big tau occurs at the late stages of neuronal differentiation [9]. In rodent models, sensory neurons of the DRG and autonomic neurons of the SCG initially express only LMW tau during embryonic development. As development progresses postnatally, there is a coordinated shift toward Big tau expression, with the transition completed by 4–5 weeks after birth [10].

While Big tau is predominantly a peripheral protein, it is also expressed in select CNS regions as shown in Table 4, most notably in the cerebellum [46] and retinal ganglion cells (RGCs) where both LMW tau and Big tau are expressed in adult neurons. Interestingly, RGCs express a unique variant of Big tau that includes exon 4a and exon 6 but excludes the near-N-terminal exons 2 and 3, resulting in a protein of approximately 90 kDa [12]. This suggests that the projection domain can be finely tuned to meet the specific requirements of the ocular transport system, which may prioritize different membrane interactions compared to the longer axons of the sciatic nerve [47].

## 5. Big Tau in Non-Neuronal Physiology

Although Big tau has traditionally been regarded as a neuron-specific cytoskeletal protein growing evidence suggests that tau isoforms including Big tau also participate in broader non-neuronal physiological processes [6,48,49,50]. Non-neuronal microtubules are frequently radial, dynamically reorganized, and tightly integrated with actin and intermediate filament networks. Consequently, Big tau in non-neuronal cells may function more broadly as a regulator of local cytoplasmic viscosity, organelle tethering, cytoskeletal flexibility, and mechanical coupling rather than as a simple microtubule spacer Table 5. Indeed, tau protein and MAPT transcripts have been identified in the heart, lung, intestine, kidney, skeletal muscle, retina, skin, endocrine tissues, and enteric nervous system [51,52] although the precise isoforms expressed in many of these tissues remain incompletely characterized with the presence of autonomic innervation, which expresses Big tau in these organs may lead to false positive results.

For example, epithelial and secretory cells of the lung and gastrointestinal tract depend heavily on polarized intracellular trafficking systems that shuttle vesicles and membrane proteins between apical and basal compartments. In these cells, Big tau-like isoforms may organize transport corridors within crowded cytoplasmic environments rather than maintaining stable long-range axonal tracks. The enteric nervous system (ENS) is especially relevant because enteric neurons undergo lifelong remodeling in response to microbiome composition, diet, inflammation, and autonomic signaling. Tau proteins within the ENS may therefore contribute to cytoskeletal resilience and sustained transport capacity under chronic metabolic and inflammatory stress. In another example, Big tau has been found in mammalian heart, where it may play a role in myocardial stiffness and excitation-contraction coupling [53]. By regulating the tyrosination state of cardiac microtubules, Big tau may support proper myocardial elasticity and diastolic function. Disruption of Big tau in this study was associated with heart failure and myocardial stiffness, reinforcing the idea that the 250-amino acid insert is a universal requirement for structural stability in high-mechanical-stress environments. These observations support a broader evolutionary interpretation of Big tau function. Rather than evolving solely as a neuronal molecule, Big tau may represent a versatile solution for maintaining cytoskeletal organization in large, long-lived, mechanically stressed vertebrate cells. As vertebrates evolved larger body plans, increasingly complex autonomic systems, and greater transport demands, we hypothesize that selective pressure may have favored tau isoforms capable of generating larger steric exclusion zones and enhanced proteostatic buffering. We suggest that under this framework, the importance of Big tau derives less from precise amino acid sequence and more from emergent physical properties such as length, charge density, hydration volume, and conformational flexibility. From a translational perspective, understanding Big tau in both neuronal and non-neuronal systems may provide important insights into neurodegeneration and systemic aging. Peripheral tissues are increasingly recognized as contributors to neurodegenerative disease progression through inflammatory signaling, extracellular vesicle trafficking, and protopathic seeding. If Big tau indeed possesses intrinsic anti-aggregation and cytoskeletal-buffering properties, engineering analogous structural features into CNS tau isoforms could represent a promising therapeutic strategy for Alzheimer’s disease and related tauopathies.

## 6. General Lessons from the Analysis of 4a Properties and Evolution

### 6.1. The Mechanism of the Entropic Bristle and Microtubule Spacing

To understand why exon 4a lengths cluster around approximately 250 amino acids, we must go deeper into the biophysics of entropic forces and the molecular environment of the axon. The function of a molecular spacer is to provide a resistive force against compression. In the case of Big tau, this force is generated by the conformational entropy of the disordered 4a insert [19,23].

A disordered polypeptide like exon 4a does not have a static shape; it behaves like a “cloud” of potential states. When two microtubules decorated with Big tau are pushed together, the available space for these “clouds” decreases. This reduction in volume limits the number of possible conformations the protein can adopt, thereby lowering its entropy. 

The 250-amino acid length is the evolutionary “sweet spot” for this pressure. Shorter sequences (LMW tau) generate enough pressure to maintain a 20 nm gap, which is sufficient for small-caliber brain axons. However, the large-diameter axons of the PNS require a 35 nm gap to accommodate mitochondrial clusters and large vesicular cargo. A domain significantly longer than 250 residues would increase this gap further but might become energetically costly to translate or create excessive “drag” during transport. Thus, the conservation of size represents an optimization of axonal geometry against metabolic cost.

### 6.2. The Polyelectrolyte Brush Model

The strongly acidic composition of exon 4a adds an electrostatic component to this repulsive force. Analyses of exon 4a sequences demonstrate enrichment in negatively charged residues together with high hydrophilicity and low β-sheet propensity [13,37]. Because neighboring Big tau molecules carry similar negative charges, electrostatic repulsion promotes extension of the projection domains away from the microtubule lattice into a “polyelectrolyte brush” configuration [54,55,56]. This effect increases the effective reach of the entropic bristle and enhances microtubule spacing even at relatively low surface occupancy as summarized in Table 6. Experimental force measurements and structural studies strongly support polymer-brush and polyelectrolyte-brush models for tau-mediated microtubule organization.

### 6.3. Genomic Hotspots for Tau Expansion

MAPT appears unusually permissive to alternative splicing changes and retained-intron isoforms. Beyond exon 4a, several species exhibit other high-molecular-weight tau variants, such as the inclusion of exon 6 in certain rodent cell lines and the human-specific W-tau arising from intron 12 retention [44,57] as well as intron retention of exon 11 [58,59]. This pattern of periodic genomic expansion suggests that the biological demand for an elongated tau projection domain has recurred throughout vertebrate history.

In primates, the stabilization of exon 4a-L (355 residues) was likely driven by the recruitment of primate-specific transposable elements like Alu SINEs [60]. These elements are rich in “cryptic” splice sites that can be activated by single-nucleotide mutations. If a bird or an amphibian faced the same structural requirement—the need for a 250-amino acid spacer—but lacked Alu elements, its genome would have “exonized” whatever material was available in the intronic regions of its ancestral MAPT gene. This would result in the current observation of a conserved physical dimension (the length required for microtubule spacing) derived from entirely different genomic building blocks (different transposable elements or intronic sequences).

Distinguishing the prototype and independent exonization models will require using comparative genomics, developmental expression, and functional analyses. The prototype model predicts that exon 4a originated from an ancestral sequence that retained a conserved function, whereas independent exonization predicts repeated, lineage-specific recruitment of intronic sequence with convergent functional properties. Comparative analysis of MAPT gene across a large range of species, including sequence conservation, splice-site architecture, and intron retention of exon 4a, should therefore provide important evidence. Long-read transcriptomics and developmental RNA sequencing could further determine whether exon 4a-like sequences arise through alternative exonization or intron retention and whether their expression is independently regulated.

The evidence of regulatory plasticity of MAPT which can tolerates major alternative splicing changes during evolution is consistent with both exonization and intron retention. We therefore highlight the differences between these mechanisms in Table 7.

### 6.4. The Evolution of Intrinsically Disordered Regions (IDRs)

The case of exon 4a highlights the unique evolutionary rules governing intrinsically disordered proteins [4,15,39]. Traditional protein evolution models, such as the PAM (Percent Accepted Mutation) and BLOSUM (Blocks Substitution Matrix), are optimized for structured domains where amino acid substitutions are constrained by the need to maintain a hydrophobic core or a catalytic site [61]. IDRs, however, evolve under a different set of constraints. In IDRs like exon 4a, selection often targets “distributed biophysical features” rather than primary sequence identity. These features include: Hydropathy: Maintaining high solubility to prevent misfolding, Net Charge: Ensuring long-range electrostatic repulsion, Flexible Disorder: Preserving the entropic bristle function, and Length: Determining the physical reach of the repulsive force.

Because many different sequences can satisfy these requirements, IDRs like exon 4a can accumulate mutations at a much higher rate than structured domains. This explains the “accelerated drift” observed in Big tau; as long as the domain remains ~250 residues long and highly acidic, the specific sequence of amino acids can drift toward near-random combinations without compromising function.

Although the 4a domain is predicted to favor disorder rather than stable secondary structure, it is not yet known whether particular sequence motifs or structural features are required, or whether their disruption produces loss- or gain-of-function phenotypes. Unlike other regions of MAPT, there are currently no well-established pathogenic variants specifically within exon 4a that have been clearly linked to inherited neuropathy or tauopathy. Isoform-specific knock-in, deletion, and mutagenesis models in long-axon neurons could determine whether particular sequence features are required for Big-tau-dependent microtubule organization, axonal transport, or neuronal survival, and whether disruption preferentially affects long-projecting neurons.

### 6.5. Hidden Sequence Patterns and Convergent Phenotypes

Recent computational analyses using machine-learning–based protein sequence models have suggested that highly diverged IDRs may still preserve subtle organizational principles or “hidden” biophysical signatures [62]. Although exon 4a sequences exhibit little conventional sequence conservation, the maintenance of characteristic charge distributions and disorder-promoting composition suggests that selection acts at the level of ensemble biophysical behavior rather than local sequence motifs. These observations support the concept that the evolution of Big tau reflects conservation of a physical phenotype defined by molecular reach, electrostatic repulsion, and spacer function across vertebrate evolution [13,37].

## 7. Clinical Implications

### 7.1. The Shielding Deficit in the Aging Brain

The evolutionary “shield” provided by exon 4a may have important implications for therapeutic strategies targeting neurodegeneration. Most research on tauopathies and Alzheimer’s disease has focused on eliminating tau aggregates after their formation [63,64,65]. However, the biology of Big tau suggests a potentially complementary preventative strategy based on restoring the protein’s physical shielding properties.

One hypothesis is that the vulnerability of cortical neurons to tau aggregation reflects a relative “shielding deficit.” CNS neurons predominantly express low-molecular-weight (LMW) tau isoforms with relatively short projection domains [35]. These shorter domains may provide insufficient steric and electrostatic separation between tau molecules, allowing aggregation-prone motifs such as PHF6 to interact more readily under conditions of aging, oxidative stress, or abnormal phosphorylation [22,66]. In contrast, mature peripheral neurons express Big tau containing the large exon 4a projection domain, which may function as a persistent entropic and electrostatic barrier limiting intermolecular tau interactions [13]. This interpretation is consistent with the long-recognized observation that peripheral nervous system neurons are comparatively resistant to tau aggregate pathology. The structural and tissue-specific properties of Big tau may therefore provide important insights for both diagnostic and therapeutic development.

### 7.2. Designing Synthetic Shields for the CNS

**Figure 6:**
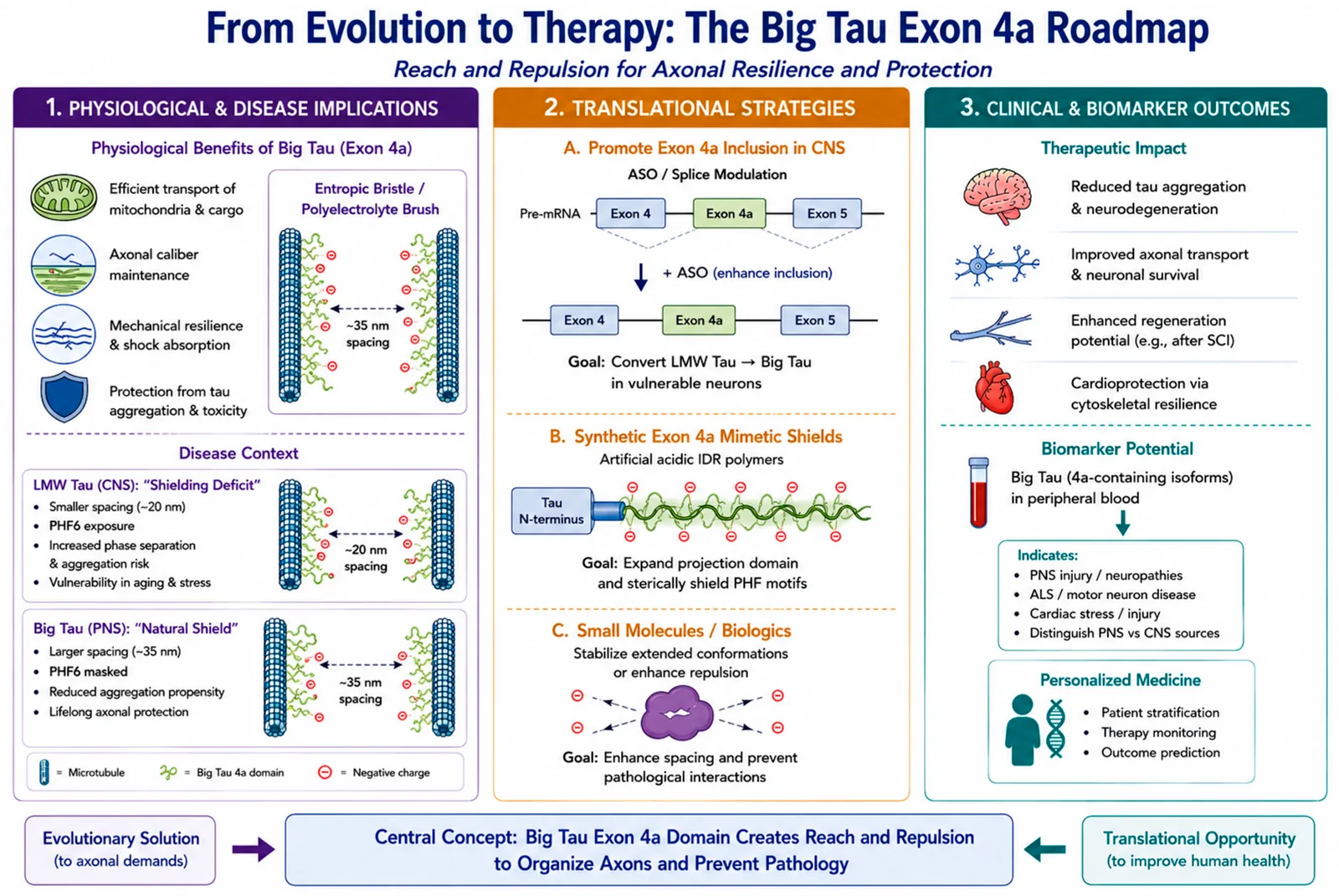
Clinical implications. The figure summarizes the protective properties of Big tau and the translational strategies that include gene therapy, use of ASO, biomimetic shields to prevent aggregation, and the use of Big tau as a biomarker.

The apparent protective effect of the exon 4a projection domain provides a conceptual framework for a new class of tau-directed therapeutics (Figure 6). If inclusion of exon 4a reduces aggregation susceptibility, gene therapy or antisense oligonucleotide (ASO) approaches could potentially be used to promote exon 4a inclusion in vulnerable CNS neurons [67,68,69]. Such strategies would aim to convert LMW tau into Big tau-like isoforms by expanding the tau projection domain and restoring steric shielding around aggregation-prone regions. ASO-mediated splice modulation has already shown therapeutic success in neurological diseases such as Spinal Muscular Atrophy, demonstrating the feasibility of manipulating alternative splicing in the nervous system [67]. Similar approaches could theoretically be adapted to the MAPT locus to favor exon 4a incorporation in hippocampal and neocortical neurons. It can also be used to promote neuroprotection and regeneration after injury particularly spinal cord injury where brain connectivity is disrupted. Increasing Big-tau expression with ASOs should be approached cautiously. The absence of Big tau from many CNS neurons could itself be biologically important. Forced exon 4a inclusion could therefore alter tau abundance, stoichiometry among tau isoforms, microtubule interactions, or axonal transport, with potentially unpredictable consequences in neurons that do not normally express Big tau. In addition, increasing Big tau in a tauopathy could be problematic if the disease is driven by tau accumulation or altered tau proteostasis, even if the Big-tau isoform itself is not intrinsically pathogenic.

This strategy differs fundamentally from conventional tau-lowering approaches because it addresses the physical determinants of aggregation rather than simply reducing tau abundance or removing existing fibrils [64,70]. In parallel, synthetic biomaterials or engineered intrinsically disordered polymers with high negative charge density could potentially mimic the entropic shielding properties of exon 4a by binding the tau N-terminus and sterically masking PHF6-containing aggregation cores [22,71].

### 7.3. Exon 4a as a Biomarker for PNS Disorders

The highly restricted distribution of Big tau may also offer opportunities for biomarker development. One major challenge in blood-based tau diagnostics is distinguishing CNS-derived tau from peripheral tau species [72]. Because Big tau expression is enriched in the peripheral nervous system and relatively absent from most cortical neurons, circulating Big tau may provide a more selective indicator of peripheral axonal injury [73].

Alterations in Big tau concentration or phosphorylation state could reflect peripheral neuropathy, axonal degeneration, traumatic nerve injury, or motor neuron disease such as Amyotrophic Lateral Sclerosis (ALS). Similarly, because Big tau is also expressed in select non-neuronal tissues including cardiac tissue, changes in Big tau-related biomarkers might reflect broader cytoskeletal stress responses.

Although these possibilities remain largely unexplored experimentally, the tissue specificity and unusual structural features of Big tau make exon 4a–containing isoforms attractive candidates for next-generation peripheral neurodegenerative biomarkers.

## 8. Final Synthesis: The Evolutionary Legacy of Big Tau

The evolution of the Big tau 4a exon stands as a compelling example of physical geometry driving genomic selection similar to other proteins such as titin in muscles [74]. Across the vast span of vertebrate evolution, the composition details of the domain have been sacrificed to the relentless drift of neutral mutation, yet the macroscopic properties—a length of approximately 250 residues and a high density of negative charge—have been preserved with extraordinary fidelity. Exon 4a is essentially a molecular machine defined by its volume and its reach. It serves as an entropic anchor for the axonal cytoskeleton, ensuring that microtubules are held at the 35 nm distance required for the transport of the largest cellular cargoes in high-caliber projection neurons. Simultaneously, this expanded reach provides a “proteostatic shield,” limiting the exposure of aggregation-prone motifs and explaining why Big-tau-expressing neurons are spared from the ravages of tauopathy. Whether exon 4a originated through an ancient ancestral prototype or via the opportunistic and repeated exonization of transposable elements remains a subject of speculation. However, its biological necessity is clear. As vertebrates evolved more complex motor systems and longer bodies, the MAPT locus repeatedly expanded to provide the structural support required for such complexity. By moving toward a new paradigm of protein evolution, one where physical ensemble properties are as important as the amino acid sequences, we can begin to fully appreciate the evolutionary pathway that produced the Big tau protein and utilize its protective properties to combat neurodegenerative disease. Leveraging these “protective secrets” offers a possible strategy for neurodegenerative medicine, providing the tools to utilize the resilience of the peripheral nervous system to fortify the aging brain against neurodegeneration.

Despite the progress in investigating the biophysical and evolutionary properties of Big tau we need to consider the current limitations. Our interpretation of exon 4a as a conserved biophysical spacer relies largely on comparative sequence analysis, predicted disorder, charge distribution, and inferred polymer behavior rather than direct functional testing. Consequently, the observed stability of exon length and its biophysical properties do not by itself distinguish between an ancestral prototype model, convergent exonization, or alternative constraints imposed by exon architecture and splicing. Likewise, the proposed protective role of exon 4a against tau aggregation is sound but may reflect indirect effects on localization, phosphorylation accessibility, or proteostasis rather than direct steric shielding alone.

## 9. Conclusions

The conserved size and low sequence identity of exon 4a, the defining feature of Big tau, were examined from the perspectives of genomic architecture and biophysical properties. This analysis supports two plausible evolutionary scenarios: a prototype model in which an early exon insertion underwent largely neutral sequence drift while preserving length, and independent exonization of non-coding DNA through transposable element insertion or intron retention, driven by convergent evolutionary recruitment. The proposed functions of Big tau as a molecular spacer that optimizes microtubule organization in long-projection neurons and as a structural feature that may protect against pathological tau aggregation remain working hypotheses that will require validation through future in vitro and in vivo studies.

## Figures and Tables

**Table 1:** Sequence comparisons of the 4a exon in vertebrates.

Organism	Species	Exon 4a Length (aa)	Sequence Identity Relative to Human (%)
Homo sapiens	Human	251	100%
Pan troglodytes	chimpanzee	251	99%
Rattus norvegicus	Rat	254	56%
Mus musculus	Mouse	253	56%
Taeniopygia guttata	Zebra Finch	279	24%
Xenopus tropicalis	Frog	226	16%
Danio rerio)	Zebrafish	280	24%

Table 1 Sequence comparison among vertebrates show extreme divergence with a stable size. Lengths and identities were derived from the alignments reported in [7] and mapped to Ensembl annotation.

**Table 2:** Biophysical properties of 4a.

Biophysical Property	LMW Tau (Human)	Exon 4a (Human)	Big Tau (Human)
Sequence Length (aa)	525	251	776
Negative/Positive Ratio	1.11	1.3	1.18
Kyte–Doolittle Hydropathy	−0.893	−0.9259	−0.9036
Predicted b-sheet	17.33%	4.78%	13.14%
Predicted Disorder	68.19%	96.01%	78.47%
Diffusion	High	-	20–30% slower

Table 2: The table shows data that define the biophysical properties of the 4a exon being negatively charged (acidic), hydrophilic, with low level of β-sheet structure and high predicted disorder. These properties affect Big tau relative to LMW tau. The values derived from [7].

**Table 3:** Comparison of the Models.

Feature	Prototype Model	Independent Exonization
Origin	Single ancestral event	Multiple lineage-specific events
Explanation for Low Sequence Identity	Maximum neutral drift in a flexible IDR	Different starting genomic material (introns/TEs)
Selective Pressure	Maintaining ensemble properties (length/charge)	Architectural necessity for axonal structure
Big Tau Properties	Conserved biophysical properties across classes	Distinct sizes in different species frogs (226 aa) vs. humans (251 aa); 4a-L (355 aa) variant [7]

Table 3. The table compared the two models with respect to known molecular and evolutionary mechanisms [13,45].

**Table 4:** Tau properties and distribution.

Tissue Type	Predominant Tau Form	Exon Composition	Apparent MW
CNS (e.g., cortex)	LMW Tau	No 4a, variable 2/3/10	45–65 kDa
PNS (e.g., DRG)	Big Tau	4a, 6	110kDa
CNS (RGC)	Big Tau (low levels)	4a, 6, skipping 2/3	90 kDa
Heart	Big Tau?	4a, 6?	110 kDa
Cancer cells	Big tau variant	4a-L	>110 kDa

Table 4: The table summarize the composition and distribution of LMW tau and Big tau with their known variants [35].

**Table 5:** Big tau properties and distribution in neurons and peripheral organs.

Neurons	Peripheral Organs
Maintaining microtubule organization	Cytoplasmic buffering scaffold
Increase spacing of microtubules	Stabilizer of organelle positioning
Facilitating axonal transport	Mechanical shock absorber
Provide protection against aggregation	Anti-aggregation or proteostatic factor

Table 5. The table compares what is known about the properties of Big tau in neurons (mostly PNS) relative to what we hypothesize their function may be in peripheral organs, where much less in known and where the specific function may dependent on the type of tissue in any organ [48].

**Table 6:** The Polyelectrolyte Brush Model.

Spacer Parameter	LMW Tau	Big Tau (Exon 4a)
Predominant Environment	Cortical Axons	Sciatic Nerve/Heart
Repulsive Gap	~20 nm	~35 nm
Charge Density	Moderate	High Negative
Entropic Reach (R_G_)	~3–4 nm	~5.5–6.7 nm
Shielding Capacity	Low	High

Table 6. The electrostatic properties of LMW tau and Big tau are model-derived estimates in the context of the polyelectrolyte model [13,45].

**Table 7:** Exonization vs. intron retention mechanism.

Feature	Exonization	Intron Retention
Definition	A sequence that was not an exon is included as a new exon.	An intron that would normally be removed remains in the mature transcript.
Outcome	Adds a new exon to the mRNA.	Keeps intronic sequence in the mRNA.
Source	Intron or transposable element	A normally spliced intron.
Coding effects	Can add new amino acids or create new protein features.	Can introduce stop codons, change translation, trigger RNA decay.
Mechanism	Often evolutionary or regulatory; can also be disease-associated.	Often regulatory, developmental, stress-responsive, or disease-associated.
Tau example	Big tau/exon 4a is the clearest tau-related example.	W-tau and related intron-retained MAPT are the clearest tau-related examples.
Key concept	“A new exon is created.”	“An intron is kept.”

Table 7: structural and functional differences between exonization and intron retention.

## Data Availability

No new data were created or analyzed in this study. Data sharing is not applicable to this article.
